# Two-Year Follow-Up on Chemosensory Dysfunction and Adaptive Immune Response after Infection with SARS-CoV-2 in a Cohort of 44 Healthcare Workers

**DOI:** 10.3390/life12101556

**Published:** 2022-10-07

**Authors:** Sophia E. Schambeck, Laura M. Mateyka, Teresa Burrell, Natalia Graf, Ioana Brill, Thomas Stark, Ulrike Protzer, Dirk H. Busch, Markus Gerhard, Henriette Riehl, Holger Poppert

**Affiliations:** 1 Helios Klinikum Munich West, Steinerweg 5, 81241 Munich, Germany; 2 Institute for Medical Microbiology, Immunology and Hygiene, School of Medicine, Technical University of Munich, Trogerstr. 30, 81675 Munich, Germany; 3 Institute of Virology, School of Medicine, Technical University of Munich, Trogerstr. 30, 81675 Munich, Germany; 4 German Center for Infection Research (DZIF), Partner Site Munich, 81675 Munich, Germany; 5 Klinik und Poliklinik für Neurologie im Neuro-Kopf-Zentrum, Klinikum rechts der Isar, Ismaninger Str. 22, 81675 Munich, Germany

**Keywords:** parosmia, phantosmia, dysgeusia, hyperosmia, smell, taste, SARS-CoV-2, long-COVID, quality of life, immune response

## Abstract

Persistent chemosensory dysfunction (PCD) is a common symptom of long-COVID. Chemosensory dysfunction (CD) as well as SARS-CoV-2-specific antibody levels and CD8^+^ T-cell immunity were investigated in a cohort of 44 healthcare workers up to a median of 721 days after a positive PCR test. CD was assessed using questionnaires and psychophysical screening tests. After 721 days, 11 of 44 (25%) participants reported PCD, with five describing an impaired quality of life. One participant reported hyperosmia (increased sense of smell). The risk of PCD at 721 days was higher for participants reporting qualitative changes (parosmia (altered smell), dysgeusia (altered taste), or phantosmia (hallucination of smell)) during initial infection than in those with isolated quantitative losses during the first COVID-19 infection (62.5% vs. 7.1%). The main recovery rate occurred within the first 100 days and did not continue until follow-up at 2 years. No correlation was found between antibody levels and CD, but we observed a trend of a higher percentage of T-cell responders in participants with CD. In conclusion, a significant proportion of patients suffer from PCD and impaired quality of life 2 years after initial infection. Qualitative changes in smell or taste during COVID-19 pose a higher risk for PCD.

## 1. Introduction

Sudden loss of taste (ageusia) and smell (anosmia) are distinctive and frequent symptoms of the acute phase of Coronavirus Disease 2019 (COVID-19) [1,2,3]. The prevalence of self-reported chemosensory dysfunction (CD) during the illness was up to 86% for European patients infected in 2020 and decreased to around 30% or less with the spreading of the Omicron variant (BA1 and BA2 lineage) of Severe Acute Respiratory Syndrome Coronavirus 2 (SARS-CoV-2) in 2022 [4,5,6].

In many cases, the patients’ function of taste and smell significantly improves within the first weeks after symptom onset, and the prevalence of complete quantitative losses of smell and taste drops [7,8,9]. In contrast, the prevalence of qualitative dysfunctions, such as phantosmia (hallucination of smell), parosmia (alteration of smell), and dysgeusia (alteration of taste) rises over time due to an often-delayed onset of several months [8,10,11,12]. Long-term observation studies commonly report that a substantial number of patients seem not fully recovered from SARS-CoV-2-caused chemosensory dysfunction up to 24 months post-infection [9,10,13]. Persistent chemosensory dysfunction (PCD) is among the most frequently reported symptoms of long-COVID [12]. COVID-19-related dysfunctions of smell and taste, especially qualitative changes, can disrupt eating habits and severely affect the quality of life [14,15,16]. In addition, PCD after COVID-19 could be an indicator of an increased risk to develop neurodegenerative diseases [17,18]. Therefore, it is important to learn more about the course of recovery and the long-term outcome of SARS-CoV-2-induced dysfunction of smell and taste.

Only a few studies report on PCD two years after infection, showing varied results with a self-reported prevalence of 11–61% [13,19,20,21]. Since self-reports tend to poorly correlate and often underestimate psychophysically measured chemosensory dysfunction, it is important to additionally apply psychophysical testing [22,23]. So far, only one other study reports on psychophysically tested chemosensory function after two years [19].

To fill this gap this follow-up study evaluated self-reported and psychophysically measured chemosensory function in a cohort of 44 health workers, at a median of 721 days after the first positive SARS-CoV-2 PCR test result. Participants showed no or only mild symptoms of COVID-19 and were not hospitalized during the illness. Participants’ results after a median of 721 days were compared to those after a median of 100 and 244 days. Antibody levels and CD8^+^ T-cell response were measured to investigate possible correlations between immune response and alterations in taste and smell.

## 2. Materials and Methods

### 2.1. Participants

In this follow-up study, data were obtained from 44 subjects. The cohort included 40 hospital employees, who acquired the first PCR-confirmed SARS-CoV-2 infection at Helios Clinic Munich West in 2020. Four direct relatives, infected by their partners, were also included in the study. All participants completed the initial substudy on olfactory and gustatory function after the first infection with SARS-CoV-2 [11]. Participation was voluntary and not remunerated.

Inclusion criteria were a positive RT-PCR test for SARS-CoV-2 and age 18 years or older. Patients with olfactory or gustatory dysfunctions known prior to the onset of the pandemic were excluded.

### 2.2. Study Design

**Figure 1:**
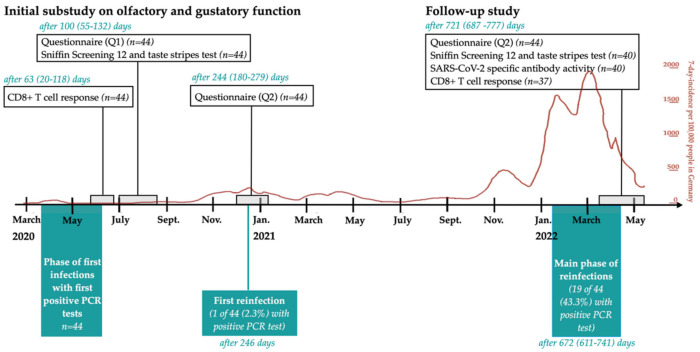
Timeline showing the elements of the initial substudy and the follow-up study, as well as phases of infections and reinfections in the cohort. Time points are presented as median (range) days post first positive PCR test. Forty-four individuals were included in the study and took part until 721 days after the first infection. The Sniffin Screening 12 test was applied after 100 and 721 days, and questionnaire Q2 after 244 and 721 days. In addition, SARS-CoV-2 specific antibody activity and CD8^+^ T-cell response were measured at different time points. The seven-day-incidence per 100.000 people in Germany over the course of the study is visible on top of the timeline [19].

This monocentric, prospective follow-up study investigated psychophysical and subjective olfactory and gustatory function as well as an adaptive immune response at a median of 721 days post first positive SARS-CoV-2 PCR test result. This study represents the second follow-up of the study “Establishment and validation of epitope-specific SARS-CoV-2 blood-based testing methods” (EPI-SARS). In the first follow-up substudy, self-reported and psychophysically tested olfactory and gustatory functions were evaluated at a median of 100 and 244 days post-infection (see Figure 1). CD8^+^ T-cell response was analyzed after a median of 63 days.

Interlinks with EPI-SARS as well as results, methods, and a detailed description of T-cell response from the previous substudy are published elsewhere [11,24].

From February 2022 onwards, participants were invited to take part in the follow-up study. Within one study visit, participants completed the Sniffin Screening 12 and Taste stripes test, questionnaire Q2, an additional structured questionnaire concerning the quality of life, reinfections with SARS-CoV-2 and vaccination status, and donated serum and full blood samples. Participants who could not complete the follow-up in person were asked to answer the questionnaires on the telephone. Study visits were scheduled as close as possible to the two-year mark after the first positive SARS-CoV-2 RT-PCR test result. Figure 1 shows the time points and structure of the initial substudy and follow-up study visits, as well as phases of first infections and reinfections with SARS-CoV-2 within the cohort. As hospital employees, the participants were screened for reinfection with SARS-CoV-2 by PCR tests on a regular basis.

### 2.3. Questionnaires Q1 and Q2

The self-constructed structured questionnaires Q1 and Q2 addressed olfactory and gustatory function at the time of the study visits as well as during acute COVID-19 infection, retrospectively. Q1 was applied after a median of 100 days, Q2 after a median of 244 and 721 days. Both questionnaires have been previously described in detail [13].

Q1 measured self-reported smell and taste function 100 days after infection as well as retrospectively during acute COVID-19 infection. Participants were asked if they had experienced dysfunction in taste or smell at the beginning of the illness and if they still noticed any changes in taste. The same questions were applied to smell. Furthermore, they were asked if they currently noticed anything unusual in their sense of smell or taste. This open question revealed qualitative dysfunctions in several participants which led to the development of questionnaire Q2.

In Q2, subjects were asked to evaluate their current olfactory and gustatory function. The answer options for taste as well as for smell were: “as before”, “changed”, “hallucinatory” or “diminished”. In addition, participants were asked to describe again their chemosensory dysfunction during their first COVID-19 episode in 2020. Changes in quality were assessed by the questions “Were certain smells changed?” (“no”, or if “yes” was selected, participants would describe which smells were changed and in which way), “Did you smell anything that wasn’t there? (Did you hallucinate smell(s)?)” (“no”, or if “yes”, were these hallucinated smells known, unknown, pleasant, or unpleasant?). Gustatory dysfunction during COVID-19 was assessed by the question if participants had experienced dysfunction in taste during the acute illness (“no”, or if “yes” was selected participants would further specify with the options “diminished”/“changed”/“hallucinated” and in addition describe the sensation).

### 2.4. Questionnaire Concerning Vaccination, Reinfections with COVID-19, and Quality of Life

In an additional structured questionnaire, subjects provided their vaccination status and positive SARS-CoV-2 PCR test dates. In cases of a PCR confirmed reinfection with SARS-CoV-2, chemosensory function during the second, third, or fourth COVID-19 episode was recorded using Q2. General symptoms during reinfections were also documented. Furthermore, subjects were asked if anything was remarkable or had changed regarding smell and taste and whether they feel that their quality of life is impaired by persistent chemosensory dysfunction.

### 2.5. Sniffin’ Sticks Screening 12 Test with Taste Stripes

For psychophysical evaluation of smell and taste the validated tests Sniffin’ Sticks Screening 12 Test with Taste stripes (Burghart Messtechnik GmbH, Holm, Germany) were used [25,26]. These screening tests were already applied during the initial study after a median of 100 days (Figure 1). During the follow-up study, 34 participants also completed the Sniffin’ Sticks Extended Test.

In the Sniffin’ Screening 12 Test, odor identification is assessed for 12 common smells with pen-like devices. Scores range from 0 to 12. Normosmia is reached by a score of 11 or 12, hyposmia by a score of 7 to 10, and anosmia by a score of 0 to 6.

To assess taste, Taste stripes with the four main taste qualities sweet, sour, bitter, and salty were placed on the tongue. Afterward, participants noted down which quality they perceived. A test result was considered abnormal if at least one of the four qualities was not identified correctly.

During the psychophysical assessment of olfactory and gustatory function, participants did not receive any feedback on their answers. At the time of testing, all participants were fully recovered from their COVID-19 infection and had already received two negative PCR test results.

### 2.6. Evaluation of SARS-CoV-2 Specific Antibody Levels

SARS-CoV-2-specific IgG and neutralizing antibody (nAb) levels were evaluated using the iFlash-SARS-CoV-2 chemiluminescence immunoassay kit (Shenzhen YHLO Biotech Co. Ltd. (Shenzhen, China)). The assays were performed on the iFLASH immunoassay analyzer (Shenzhen YHLO Biotech Co. Ltd. (China)) using the manufacturer’s protocol. Spike protein and nucleocapsid protein were used for detection. The assay procedure is described by Qian C, et al. [27]. The cutoff value for IgG was >10 AU/mL and >20 AU/mL for neutralizing antibodies.

Additionally, IgA antibody levels, directed against nucleocapsid protein, were analyzed using the recomWell SARS-CoV-2 IgA ELISA (Mikrogen, Neuried, Germany). According to the manufacturer’s instructions, samples with a concentration of <20 U/mL were assessed as negative, a concentration from 20 to 24 U/mL was counted as borderline, and samples with a concentration of ≥24 U/mL were counted as positive.

### 2.7. T-Cell Analysis

As described previously [11,24], peripheral blood mononuclear cells (PBMCs) were isolated from whole blood by density centrifugation for T-cell analyses. T cells were expanded for 12 days using peptide pool stimulation. The in-house designed peptide pool contains diverse epitopes predicted for the most common HLA class I molecules across various domains of the SARS-CoV-2 proteome. T-cell immunity was investigated via intracellular cytokine release in peptide stimulation assays.

### 2.8. Statistical Analysis

Statistical analysis was performed using R^®^ (The R Foundation for Statistical Computing, Version 2022.07.0).

Categorical data are shown in medians, absolute and relative frequencies, with the respective maxima and minima and the distribution of values. The Wilcoxon test was applied to evaluate the statistical significance of differences in the Sniffin’ Screening 12 Test score after 100 and 721 days and to investigate possible correlations between self-reported olfactory function and the Sniffin’ Screening 12 Test score. To measure the effect size, the eta squared (η^2^) test was used. The limits for the size of the effect were set with η^2^ 0.01 for a small, η^2^ 0.06 for a medium, and η^2^ 0.14 for a large effect [28]. Fisher’s exact test was applied to compare Taste stripes test results after 100 and 721 days and evaluate correlations between self-reported gustatory function and Taste stripes test results. For intergroup comparisons of antibody titers between participants with and without self-reported change in chemosensory function (persistent chemosensory dysfunction after 721 days (yes/no) and chemosensory dysfunction during the first infection (yes/no), the Wilcoxon test was applied as well. Analyses were conducted with all participants, “Group A” (participants only infected once) and “Group B” (participants with two infections and reinfection in 2022).

For all analyses except the eta squared test, the level of statistical significance was set at *p* < 0.05.

## 3. Results

### 3.1. Cohort Characteristics

All 44 participants acquired the first PCR-confirmed SARS-CoV-2 infection at Helios Clinic Munich West in Germany between 23 March 2020 and 10 June 2020 (median 1 April 2020). For characteristics of the cohort see Table 1. No participant was hospitalized during the first infection or reinfections with SARS-CoV-2.

The follow-up study took place between 14 March 2022 and 10 May 2022 (median 24 March 2022). All 44 participants completed the questionnaires. Then, 40 of 44 participants also took part in the psychophysical smell and taste screening tests and serological analysis of antibody levels. T-cell analysis was carried out on 37 participants. Figure 1 shows the timeline and parts of the initial substudy and the follow-up study. If data were missing, the participant was excluded from the corresponding analysis.

### 3.2. Self-Reported Chemosensory Dysfunction

#### 3.2.1. Chemosensory Dysfunction over the Course of the Study

In total, 30 of 44 (68.2%) participants reported chemosensory dysfunction during the first COVID-19 episode. After a median of 100 days, it was 11 of 44 (25%) and after 244 days, 14 of 44 (31.8%), which has been described in detail previously [11].

**Figure 2:**
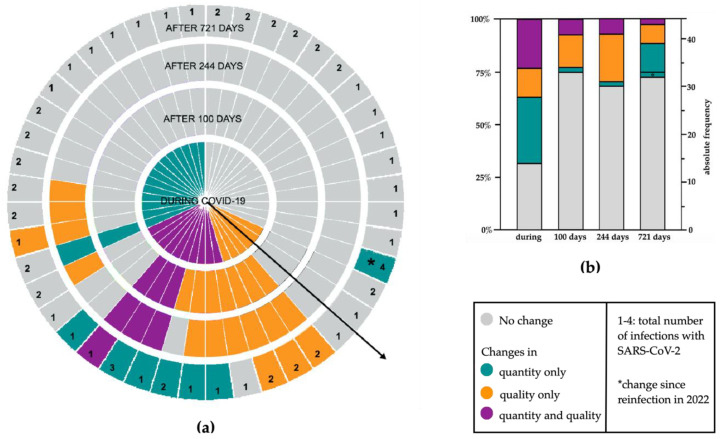
Quantitative and qualitative alteration of taste and smell of all 44 participants over the course of the study. Data on chemosensory dysfunction during the disease were collected retrospectively, whereas information concerning time points “100 days” (Q1), “244 days”, and “721 days” (Q2) were collected prospectively. (**a**) Development of quantitative and qualitative alterations of taste and smell of each participant. Each slice symbolizes one participant and the respective alteration of taste and smell during COVID-19 infection, as well as after a median of 100, 244, and 721 days. The numbers in the outer circle represent the total number of infections with SARS-CoV-2. (**b**) Absolute frequencies and percentage of qualitative and quantitative alterations of all participants during COVID-19 as well as 100, 244, and 721 days after.

After a median of 721 days, 12 of 44 (27.3%) participants reported chemosensory dysfunctions. One of these acquired hyposmia during reinfection with SARS-CoV-2 in 2022 (marked with “*” in Figure 2 and Table A1). The other 11 of 44 (25%) participants had reported chemosensory dysfunctions (7 quantitative, 4 qualitative, 1 qualitative, and quantitative) since their first infection. Figure 2 shows the participants’ development of qualitative and quantitative changes in chemosensory function over the course of the study. Table A1 shows the constellation of symptoms at different time points.

Comparing time points “244 days” and “721 days”, four participants reported the disappearance of qualitative symptoms, in four cases qualitative symptoms gave way to hyposmia and five participants reported persistent qualitative symptoms.

The comparison between participants with symptoms “during COVID-19” and after “721 days” showed that 1 of the 14 (7.1%) participants with isolated quantitative losses during the first COVID-19 episode, reported PCD (phantosmia) after 721 days. There were 10 of the 16 (62.5%) participants with qualitative changes (isolated or in combination with quantitative losses) during the first COVID-19 that reported PCD after 721 days (see Figure 2a and Table A1 for the constellation of symptoms).

#### 3.2.2. Self-Reported Olfactory and Gustatory Dysfunction during Reinfections

Overall, 20 of 44 (45,5%) participants were reinfected with SARS-CoV-2 (see Table 1 and Figure 2a). Two of the twenty (10%) reported hyposmia during reinfection. Both were asymptomatic regarding smell and taste during their first infection in 2020 and were reinfected in early 2022. One participant (marked in Figure 1 and Table A1 by “*”) reported a persistent quantitative loss after 111 days, the other had fully recovered within two weeks. None of the participants with chemosensory dysfunction during the first COVID-19 episode reported repeated chemosensory dysfunction during reinfection.

#### 3.2.3. Quality of Life and Taste and Smell after 721 Days

There were 5 of 44 (11.4%) participants (3 quantity only, 2 quality only) who reported persistent chemosensory dysfunction since their first infection, and who described a resulting impairment of their quality of life after a median of 721 days.

### 3.3. Psychophysical Screening Tests after a Median of 721 and 100 Days

#### 3.3.1. Sniffin’ Screening 12 Test

**Figure 3:**
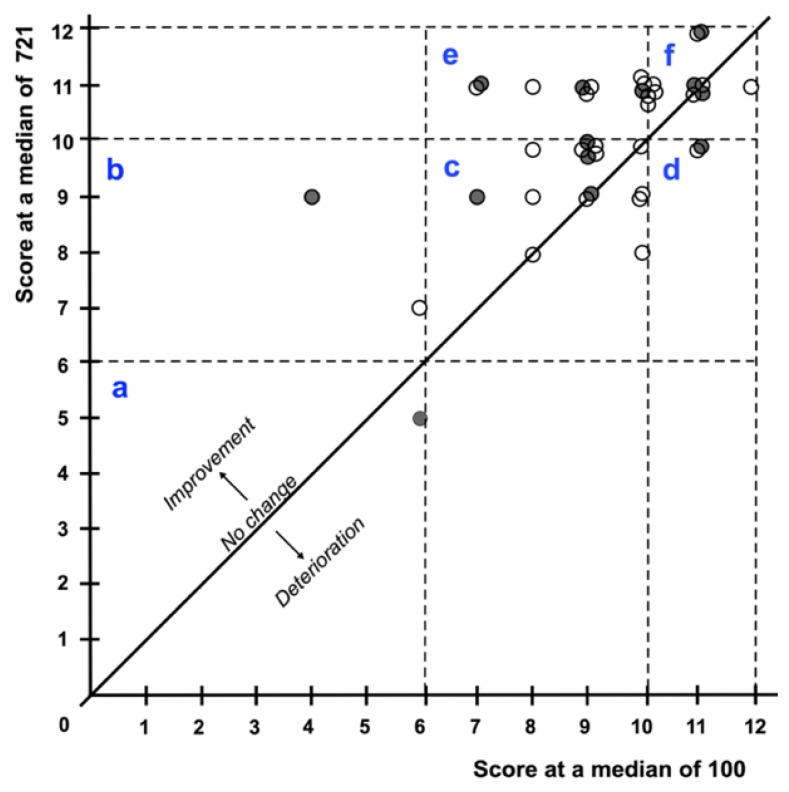
Scores of the Sniffin’ Screening 12 Test after a median of 100 and 721 days per participant (female (white circle), male (black circle)). Field a: stayed anosmic (A) (1), field b: improved from A to hyposmic (H) (2), field c: stayed H (15), field d: deteriorated from normosmic (N) to H (2), field e: improved from H to N (13), field f: stayed N (7).

**Figure A1:**
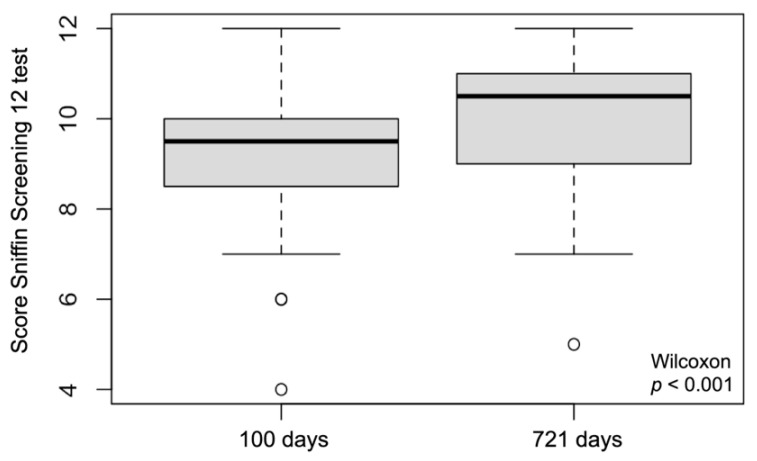
Sniffin’ Screening 12 Test scores at a median of 100 and 721 days after first positive RT-PCR test. Data analysis with the Wilcoxon test showed a significant difference between both groups (Wilcoxon *p* < 0.001).

After a median of 721 days, 20 of 40 (50%) participants reached a score of 11 or 12 and therefore normosmia. There were 19 of 40 (47.5%) participants that were within the range of hyposmia with a score of 7 to 10, and 1 of 40 (2.5%) participants reached a score of 5 and was therefore anosmic. Overall, participants scored significantly higher in the Sniffin’ Screening 12 Test after a median of 721 days in comparison to the results after a median of 100 days (Wilcoxon *p* < 0.001, see Figure A1). Figure 3 shows the scores of all participants after a median of 100 and 721 days and the change between the groups “normosmia”, “hyposmia”, and “anosmia”.

#### 3.3.2. Taste Stripes Test after 100 and 721 Days

After 721 days, 12 of 40 (30%) participants could not identify all taste qualities correctly. After 100 days, 16 of those 40 (40%) participants could not identify all taste qualities. The quality “salt” was not recognized in 7 cases, “sour” in 3, “bitter” in 5, and “sweet” in 2 cases. Comparing the results after 100 and 721 days, there was no significant difference between both time points (Fisher’s Exact *p* = 0.31).

### 3.4. Comparisons of Self-Report and Psychophysical Screening Tests after 721 Days

#### 3.4.1. Sniffin’ Screening 12 Test Score and Self-Reported Changes in Olfactory Function

**Figure A2:**
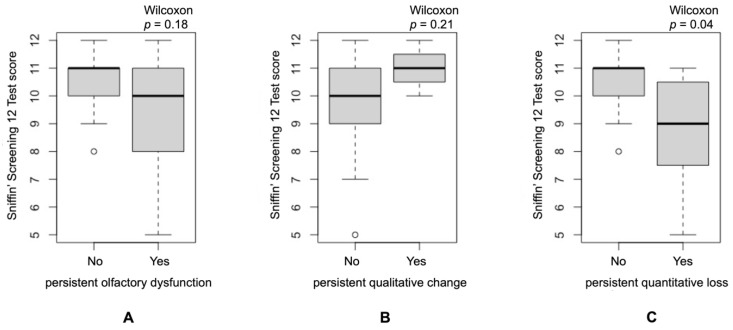
Comparisons of the Sniffin’ Screening 12 Test score of participants with or without persistent olfactory dysfunctions at a median of 721 days after the first positive RT-PCR test. (**A**,**B**) Analysis with the Wilcoxon test showed no significant difference between the score of participants with and without persistent olfactory dysfunction in general (Wilcoxon *p* = 0.18) or the score of participants with and without persistent qualitative change of smell (Wilcoxon *p* = 0.21). (**C**) Participants reporting a persistent quantitative loss of smell scored significantly lower than the asymptomatic group (Wilcoxon *p* = 0.04).

There was no significant difference in the Sniffin’ Screening 12 Test score between participants with and without self-reported persistent olfactory dysfunction (Wilcoxon *p* = 0.18) or with and without qualitative changes in smell (Wilcoxon *p* = 0.21). Participants who reported a persistent quantitative loss of smell scored significantly lower in the Sniffin’ Screening 12 Test than participants without a quantitative loss (Wilcoxon *p* = 0.04) (See Figure A2A–C).

In the eta squared (η^2^) test, self-reported persistent deficiencies in smell after a median of 721 days showed a medium effect size on the Sniffin’ Screening 12 Test score (η^2^ = 0.11), persistent quantitative loss showed a large effect on the Sniffin’ Screening 12 Test score (η^2^ = 0.21), whereas self-reported qualitative changes showed a small effect size (η^2^ = 0.04).

#### 3.4.2. Taste Stripes and Self-Reported Changes in Gustatory Function

There was no correlation between the results of the Taste stripes test and self-reported gustatory dysfunction (Fisher’s Exact *p* = 0.17), self-reported qualitative changes in taste (Fisher’s Exact *p* = 1), or self-reported quantitative losses of taste (Fisher’s Exact *p* = 0.07).

### 3.5. Self-Reported Hyperosmia after 721 Days

One participant who had reported isolated qualitative changes in taste and smell during initial COVID-19 infection, as well as at 100 and 244 days, self-reported a newly emerged higher sensitivity to all kinds of odors (hyperosmia), which had replaced persistent parosmia, after 721 days. This participant reached a score of 9 at both time points of psychophysical testing (after a median of 100 and 721 days), which is in the range of hyposmia.

### 3.6. Immune Response and Correlation with Chemosensory Dysfunction

#### 3.6.1. SARS-CoV-2 Specific Antibody Levels after 721 Days

**Figure A3:**
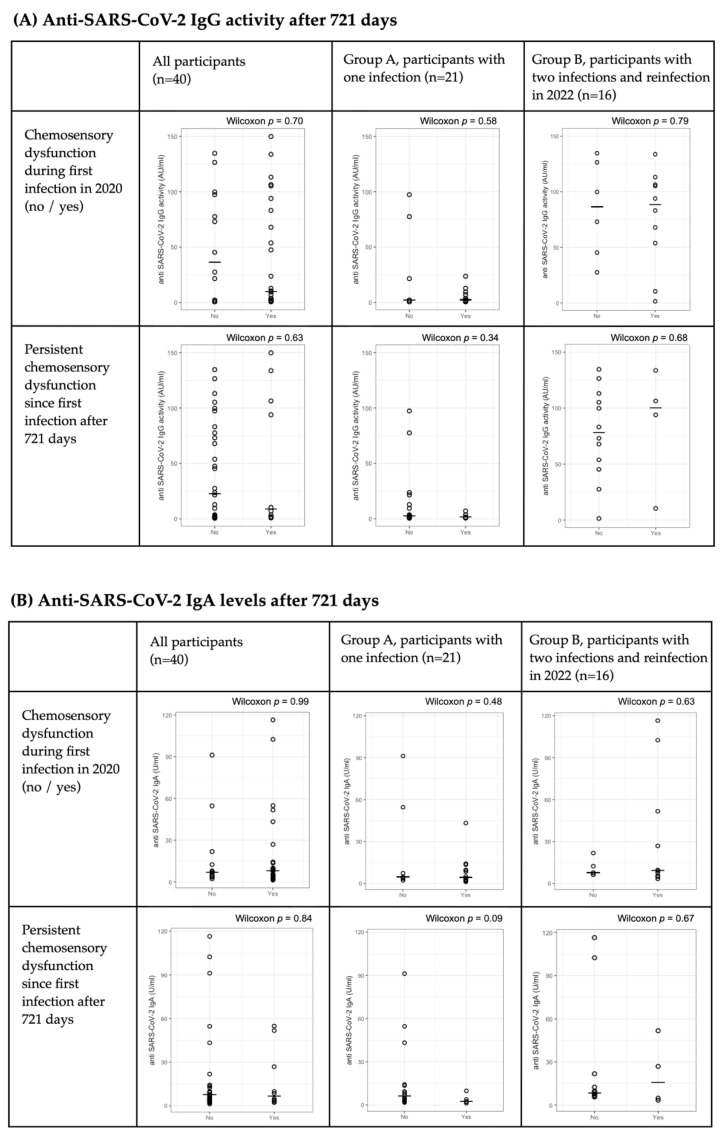
SARS-CoV-2 specific antibody levels with median after 721 days in relation to chemosensory dysfunction. The Wilcoxon test was applied for all participants (*n* = 40), group A (participants with only one infection (*n* = 21)) and group B (participants with two infections and reinfection in 2022 (*n* = 16)). Comparisons between antibody levels of participants with and without chemosensory dysfunction during the first COVID-19 episode or with and without persistent chemosensory dysfunction after 721 days showed no significant differences. (**A**) Anti-SARS-CoV-2 IgG activity and (**B**) anti-SARS-CoV-2 IgA activity after 721 days did not correlate with smell and taste symptoms.

**Figure A4:**
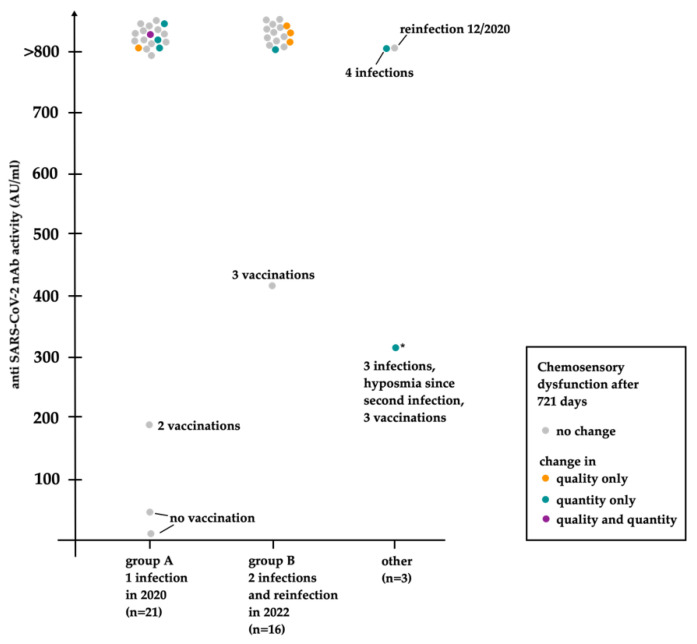
SARS-CoV-2 specific neutralizing antibody activity after 721 days in relation to chemosensory dysfunction for group A (participants with 1 infection (*n* = 21)), group B (participants with 2 infections and reinfection in 2022 (*n* = 16)), and the three remaining participants with 3 and 4 infections and reinfection in 2020. Almost all participants had nAb activity levels over 800 AU/mL. The two lowest nAb results could be explained by vaccination status. Three participants showed lower nAb levels without obvious reasons. No connection between chemosensory dysfunction and levels of nAbs was evident.

Comparisons between SARS-CoV-2 specific IgG and IgA antibody levels in participants with and without symptoms of chemosensory dysfunction at the same time point or during the first infection showed no significant difference (Figure A3A for IgG and A3B for IgA). Almost all participants had anti-SARS-CoV nAb activity at over 800 AU/mL. No connection with self-reported chemosensory dysfunction was evident. Figure A4 shows nAb levels of all participants and their respective symptoms of smell and taste after 721 days.

#### 3.6.2. SARS-CoV-2-Specific CD8^+^ T-Cell Response in Relation to Chemosensory Dysfunctions

**Figure A5:**
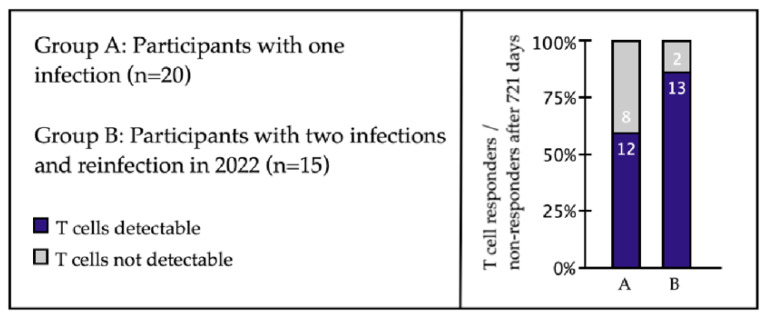
Percentage of participants with and without detectable T-cell response in “group A” (*n* = 20), with only one infection, and “group B” (*n* = 15) with two infections and reinfection in 2022.

After a median of 63 days, 34 of 44 (77.2%) participants showed a SARS-CoV-2-specific CD8^+^ T-cell response after peptide stimulation. After 721 days, 26 of 37 (70.3%) participants showed CD8^+^ T-cell responses, whereas “group A” (participants with only one infection (*n* = 20)) had a lower percentage of T-cell responders with 60% (12 of 20) than “group B” (participants with two infections and reinfection in 2022 (*n* = 15)) with 86.7% (13 of 15) (Figure A5).

**Figure A6:**
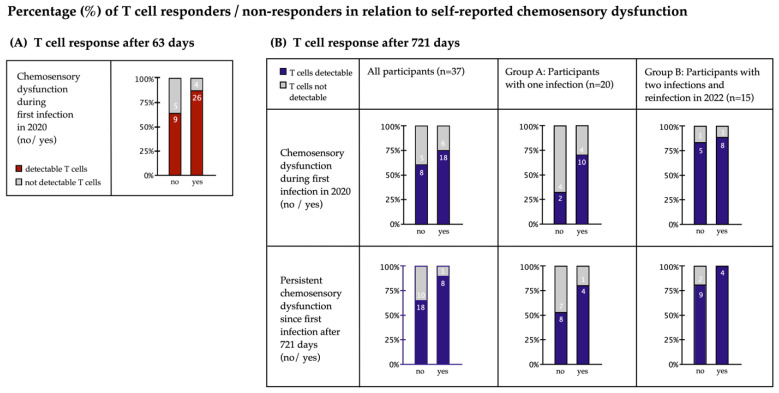
Percentage of participants with and without detectable T-cell response in relation to chemosensory dysfunction at different time points (blue and gray coloring, respectively). The number of participants in the different groups is shown in white within the columns. (**A**) Analysis of samples collected after a median of 63 days showed a higher percentage of T-cell responders in the chemosensory dysfunction (CD) group (87% with CD during vs. 64.29% without CD during). (**B**) Analysis of samples collected after 721 days showed a trend of higher responder percentages in the groups reporting CD during the first infection or PCD after 721 days. T-cell response and symptoms were analyzed for all participants (*n* = 37), group A (*n* = 20) and group B (*n* = 15). This trend was more visible in group A.

Analyzing T-cell response in relation to self-reported chemosensory dysfunctions (CD), we observed a trend for groups of participants reporting symptoms of smell or taste after 63 or 721 days to have a higher percentage of T-cell responders than groups of participants without symptoms (Figure A6A,B). After 721 days, this trend was clearer in “group A”.

## 4. Discussion

Our results show that a significant proportion of patients suffer from persistent changes in olfactory and gustatory function more than one year after infection with SARS-CoV-2, corroborating results from other studies [21,22]. Here, 11 of 44 (25%) participants reported persistent changes in smell or taste after a median of 721 days. The few other long-term observation reports, sampling 24 months after infection with SARS-CoV-2, show a self-reported persistent chemosensory dysfunction (PCD) prevalence of 11–61% [13,19,20,21]. Most of this cohort had recovered from chemosensory dysfunction within the first 100 days after the first positive PCR test. By then, 19 of the 30 (63.3%) participants who reported chemosensory dysfunction during the first COVID-19 episode reported a fully restored sense of smell and taste. Small changes were reported after a median of 244 days with the rise of late-onset qualitative dysfunctions, as described previously [11], but there were hardly any differences in terms of the patients affected at the follow-up visits after 244 and 721 days. Other studies describe self-reported or psychophysically measured main recovery rates of around 71%–80% during the first 90 days after infection [29,30,31,32], with almost no change thereafter until one year [29]. These results suggest that participants not recovering from chemosensory dysfunctions within the first few months are more likely to develop PCD, which has also been described by Fernandez et al. [33]. Nevertheless, recent studies showed that late spontaneous recovery is possible after one year [20,21]. In our cohort, patients reporting qualitative changes during the first COVID-19 episode had a higher risk of developing PCD. 62.5% (10 of 16) participants reporting qualitative changes during their first COVID-19 episode reported PCD after 721 days, whereas only 7.1% (1 of 14) with isolated quantitative loss during COVID-19 reported PCD after 721 days (Figure 2). Predictive factors for PCD are not yet established [29,34,35,36], but among others, parosmia [34] and high baseline severity of smell loss [30,32] have been associated with long recovery periods and the development of persistent olfactory dysfunctions.

In general, we observed a decreasing frequency of qualitative symptoms, such as parosmia, phantosmia, and dysgeusia comparing time points “244 days” and “721 days”. Qualitative symptoms either lifted or gave way to isolated quantitative losses (see Figure 2 and Table A1). Reden et al. describe similarly, that a significant proportion of patients with parosmia or phantosmia of various causes, recovered from these qualitative symptoms independently of improvement in quantitative olfactory capacity, within one year (29% recovery of parosmia, 53% recovery of phantosmia) [37]. Interestingly, we also observed a change in the type of qualitative dysfunction in one participant who reported parosmia and dysgeusia up to the 244-day study visit, and then at the 721-day study visit reported a newly emerged heightened sense of smell (hyperosmia), which had replaced parosmia. Hyperosmia has been described to occur during migraine attacks, after meningitis, viral infection, or toxic exposure and might be caused by uncontrolled neuronal sprouting after injury [38,39,40]. In the context of SARS-CoV-2 infection, there has been only one report of two cases with self-reported hyperosmia around two months after COVID-19 [39]. In line with the results of other cases [39,40], the hyperosmia self-reported by our participant was not evident in psychophysical test results, where hyposmia was reached. Consequently, the self-reported hyperosmia in the case reported by us stems most probably rather from hedonic hyper-perception of smell [41] than from a lowered threshold in odor detection.

The observed 25% prevalence of self-reported persistent chemosensory dysfunction two years post-infection seems to be high. Nevertheless, one must keep in mind that self-reports of chemosensory dysfunction poorly correlate with psychophysical test results and mostly underestimate the prevalence of chemosensory dysfunction [22,23,42]. Therefore, the usage of psychophysical tests is recommended to assure a more reliable assessment of chemosensory function [43,44]. This is in line with our results after 721 days. Self-reported persistent gustatory dysfunction (PGD) (8 of 40 (20%)) was less frequent than abnormal Taste stripes test results (12 of 40 (30%)) and did not correlate. The results of the Sniffin’ Screening 12 Test also showed a higher percentage of participants with an impaired sense of smell (20 of 40 (50%)) than in the self-reports (9 of 40 (22.5%)). The only other study using a psychophysical identification test two years after infection showed a low prevalence of 2.9% in the odor identification test with a higher prevalence of self-reported chemosensory dysfunction of 29,8% [19]. This might be partly explained by the olfactory training the participants of Lechien et al. completed [19]. We did not find a correlation between Sniffin’ Screening 12 Test score and self-reported PCD in general or persistent qualitative changes, but a correlation with a large effect size between self-report of quantitative losses in smell and Sniffin’ Screening 12 Test score was observed. The two participants with the lowest scores (5 (anosmia) and 7 (hyposmia)) in the Sniffin’ Screening 12 Test also reported a subjective quantitative loss. Comparing psychophysical screening test results after 100 and 721 days, there was no significant difference between the results of the Taste stripes test, but Sniffin’ Screening 12 Test scores improved significantly. Even though we only used a screening test, this result suggests that the severity of smell loss receded in our cohort, even though the number of participants reporting persistent dysfunctions stayed almost constant. Recent studies also showed a favorable development of olfactory and gustatory function and even cases of late recovery [13,19]. Similarly, Duncan et al. describe the psychophysically measured improvement of smell function in patients suffering from olfactory loss after upper respiratory infection during repeated measurements within up to five years [45]. This suggests olfactory function might also improve even years after SARS-CoV-2 infection.

There are many different explanations for the aetiologia of persistent chemosensory dysfunctions, from disruption of the olfactory epithelium to a dysregulated immune response [31,46,47]. Regarding cellular immune response, we observed a trend that participants who experienced symptoms of smell and taste at different time points had a higher percentage of CD8^+^ T-cell responders versus nonresponders after 63 or 721 days (Figure A6A,B), which was clearer in the group without reinfection. In contrast, Rank et al. did not find any correlations between T-cell response and long-term smell and taste symptoms [30]. Additional studies with larger cohorts are needed to investigate this trend. Concerning humoral immune response, we did not find any correlation between participant IgG, IgA, or nAb antibody levels and PCD after 721 days or chemosensory dysfunction during the first infection with SARS-CoV-2 (see Figure A3A,B). The literature on this topic shows varied results with some studies showing correlations between higher serum antibody levels after some months and SARS-CoV-2-induced smell and taste symptoms [48,49] and others not finding correlations [31,46]. Small cohorts, different methodologies, and varying time points of antibody titer measurement limit the interpretation of results. There is evidence that low levels of IgA [46] and salivary IgG [31] are associated with PCD. A possible explanation for this correlation is that a lack of mucosal immune response leads to chronic inflammation of olfactory tissue and consequently to PCD [46,50]. This would imply that the mucosal immune response is a key regulator of SARS-CoV-2-induced chemosensory dysfunction, whereas systemic immunity plays a minor role [51,52,53]. This thesis is supported by studies showing no significant difference in the prevalence of chemosensory dysfunction during reinfection between patients with or without vaccination [5,54], whereas chemosensory dysfunction was less likely during reinfection in participants who had previously contracted SARS-CoV-2 [54]. In contrast to natural infection, most currently available SARS-CoV-2 vaccines generate only a limited mucosal immune response [52,55]. In our cohort, 20 of 44 (25.5%) participants were reinfected with SARS-CoV-2. Only 2 (10%) reported chemosensory dysfunctions during their additional COVID-19 episode. None of the participants with chemosensory dysfunction during the first COVID-19 episode reported repeated chemosensory dysfunction during reinfection. Lechien et al. reported repeated chemosensory dysfunction during reinfections in 2020 [56,57] but even though not statistically significant, less frequent than the singular occurrence of chemosensory dysfunction [57]. Furthermore, the duration of olfactory dysfunction was significantly shorter in the cases of repeated loss during reinfection [57]. Small cohorts (*n* = 45 Lechien et al. and *n* = 44 our study) as well as different latencies between the dates of reinfection (5,6+-2,3 months Lechien et al. and around 22 months in our study) and different virus variants prevent closer comparisons and limit informative value. Larger cohorts are needed to investigate patterns of chemosensory dysfunction during reinfections.

In our cohort, we observed a low prevalence of chemosensory dysfunction during reinfection can be partly explained by the dominant virus variant during the main phase of reinfection. Except for one participant who was reinfected as early as December 2020, all were most probably reinfected with Omicron (BA.1 and BA.2 lineage), which was by far the predominant variant in Germany from January 2022 onwards [58,59]. In general, evidence is accumulating that chemosensory dysfunction occurs less frequently in more recent variants with a prevalence of only 4–33% in Omicron [5,6,54,60]. This may be due to modifications in the spike protein and herewith reduced interaction with proteins of the olfactory epithelium responsible for virus uptake, such as angiotensin-converting enzyme 2 (ACE2) and transmembrane protease, serine 2 (TMPRSS2) [61,62,63].

Although the prevalence of chemosensory dysfunction in more recent SARS-CoV-2 virus variants is declining, the number of infections and reinfections is still growing, and therefore a growing number of patients with persistent chemosensory dysfunction (PCD) can be expected. PCD of different causes can lead to reduced quality of life, which has also been described in the context of COVID-19 [12,22,64]. After around two years, 5 of 44 (11.4%) participants of this study reported an impaired life quality due to PCD. PCD seems to specifically affect the mental health component of quality of life, leaving the physical component unaffected [12]. Patients with PCD of various causes report not only depression but also eating disorders, anxiety, as well as loss of pleasure in food and social engagement [14,15,16,65,66]. The occurrence of persistent changes in smell or taste, as well as the severity of chemosensory dysfunction during COVID-19, seem to be independent of age [12,32]. Consequently, patients of all age groups can experience disruption of daily life and quality of life due to PCD. Since PCD might in addition indicate a higher risk for future neurodegenerative disease [17,18] studies on the long-term development of these symptoms should be continued.

This negative prognosis is mitigated by the long-term literature on the postinfectious olfactory loss caused by other viruses, showing that recovery occurred even years after infection [37,67]. Furthermore, olfactory training has been proven to be helpful in postinfectious olfactory loss and can induce neural reorganization processes [68,69,70,71]. Research on treatment options for chemosensory dysfunction in the context of SARS-CoV-2 is growing [19,72,73] and will become more and more important in lessening the impact of long-COVID.

## 5. Limitations and Strengths

This study’s cohort is small comprising 44 participants. Several participants were reinfected with SARS-CoV-2 and the cohort shows a variety of immunization statuses. Analysis of antibody responses in correlation with gustatory and olfactory symptoms was therefore conducted not only with all participants but also in the groups of participants with only one or only two infections to lessen the impact of this limitation. Nevertheless, the resulting singular constellations of symptoms and immunization status limit the interpretation of the study. An advantage of this cohort is, however, that all participants acquired the first infection, as well as reinfections with SARS-CoV-2 at the same place and at similar time points. In addition, the follow-up rate of 100% for self-report and around 85% for analysis of immune response after 721 days, was high. The questionnaires were self-developed, and the applied psychophysical tests are mere screening tests. Information on chemosensory dysfunction during first and repeated SARS-CoV-2 infections was collected in retrospect since our questionnaires were only applied after 100 (Q1) and 244 (Q2) days in our previous study and after 721 days (Q2) in this follow-up study. The lack of baseline measurement may have influenced the accuracy of self-reports. To lessen the impact, participants were asked repeatedly about chemosensory dysfunction during the first infection and inconsistent replies were followed up. The time points of study visits correspond to a larger range which restricts comparability between participants.

## 6. Conclusions

In conclusion, most of this cohort recovered from SARS-CoV-2-induced changes in smell and taste within the first 100 days post-infection, very little improvement was seen after this time point. After 721 days, a significant proportion (25%, 11 of 44) of participants still reported persistent chemosensory dysfunctions (PCD), with 11% (5 of 44) of the cohort reporting consequently impaired quality of life. The risk of developing PCD was higher in patients reporting qualitative changes during the first infection. There was no correlation between symptoms of smell or taste and antibody levels, but the trend of a higher percentage of CD8^+^ T-cell responders in patients with self-reported chemosensory dysfunction was observed. Furthermore, hyperosmia can occur as a late-onset symptom after SARS-CoV-2 infection.

## Figures and Tables

**Table 1:** Demographic and anamnestic information about the cohort.

Parameter	Number of Participants (%)
Sample size	44 (100%)
Gender	29 F (65.9%)
Age at date of follow-up after 721 days (median, range)	43, 24–63
Active smoker	5 (11.3%)
Number of infections with SARS-CoV-2 after 721 days	
One time	24 (54.5%)
Two times	18 (40.9%)
Three times	1 (2.3%)
Four times	1 (2.3%)
Received number of vaccinations after 721 days	
None	2 (4.5%)
One	2 (4.5%)
Two	7 (15.9%)
Three	33 (75.0%)

## Data Availability

The authors declare that the data supporting the findings of this study are available within the article and Appendix A as well as from the corresponding author upon reasonable request.

## Appendix A

**Table A1:** Reported frequencies of qualitative changes (phantosmia, parosmia, dysgeusia, and hyperosmia), quantitative losses (anosmia, hyposmia, ageusia, hypogeusia), and which sense was affected during COVID-19 as well as after a median of 100, 244 and 721 days. Data were collected by questionnaires Q1 (time points “during” and “100 days”) and Q2 (time points “during”, “244” and “721”). Information concerning smell and taste during the first COVID-19 episode was collected in retrospect, data concerning time points “100 days”, “244 days” and “721 days” were collected prospectively. Numbers marked by “*” include one participant, who had problems with hyposmia only during and since the second infection with COVID-19 in January 2022 (persistent since 111 days at follow-up study visit). All other participants suffered from the alteration in taste and/or smell since their first infection 721 days previously.

Description	During COVID-19	100 Days	244 Days	721 Days
**Asymptomatic**	**14**	**33**	**30**	**32**
**Alteration of taste or smell**	**30**	**11**	**14**	**12 ***
Quantity only	14	1	1	7 *
Taste only	1	1	0	0
*Ageusia (ag.)*	*1*	*0*	*0*	*0*
*Hypogeusia (hg.)*	*0*	*1*	*0*	*0*
Smell only	0	0	0	2 *
Taste and smell	13	0	1	5
*Ag. and anosmia (an.)*	*13*	*0*	*0*	*0*
*Hg. and hyposmia (Ho.)*	*0*	*0*	*1*	*5*
Quality only (total)	6	7	10	4
Taste only–Dysgeusia (dy.)	5	2	2	1
Smell only	0	4	3	1
*Parosmia (pa.)*	*0*	*2*	*1*	*0*
*Phantosmia (ph.)*	*0*	*1*	*2*	*1*
*Pa. and ph.*	*0*	*1*	*0*	*0*
Taste and smell	1	1	5	2
*Dy., pa. and ph.*	*1*	*1*	*2*	*0*
*Dy. and pa.*	*0*	*0*	*2*	*1*
*Dy. and ph.*	*0*	*0*	*1*	*0*
*Dy. and Hyperosmia*	*0*	*0*	*0*	*1*
Quality and quantity (total)	10	3	3	1
Taste only	0	0	0	0
Smell only-pa. and ho.	0	1	1	1
Taste and smell	10	2	2	0
*Dy. and an.*	*3*	*0*	*0*	*0*
*Dy., pa. and ph.*	*1*	*0*	*0*	*0*
*Dy., an., pa. and ph.*	*1*	*0*	*0*	*0*
*Ag., an. and pa.*	*1*	*0*	*0*	*0*
*Ag., an. and ph.*	*1*	*0*	*0*	*0*
*Ag., an., pa. and ph.*	*3*	*0*	*0*	*0*
*Dy., hg. and ho.*	*0*	*1*	*1*	*0*
*Dy., ho., pa. and ph.*	*0*	*1*	*0*	*0*
*Dy. and ho.*	*0*	*0*	*1*	*0*
